# Monoclonal Antibodies to Treat Multiple Myeloma: A Dream Come True

**DOI:** 10.3390/ijms21218192

**Published:** 2020-11-01

**Authors:** Mattia D’Agostino, Salvatore Innorcia, Mario Boccadoro, Sara Bringhen

**Affiliations:** Myeloma Unit, Division of Hematology, University of Torino, Azienda Ospedaliero-Universitaria Città della Salute e della Scienza di Torino, 10126 Torino, Italy; mattiadagostino89@gmail.com (M.D.); s.innorcia.91@gmail.com (S.I.); mario.boccadoro@unito.it (M.B.)

**Keywords:** multiple myeloma, immunotherapy, monoclonal antibodies, antibody–drug conjugates, bispecific antibodies

## Abstract

Immunotherapy is increasingly used in the treatment of multiple myeloma (MM). Monoclonal antibodies (mAbs) are safe and effective ways to elicit immunotherapeutic responses. In 2015, daratumumab has become the first mAb approved by the Food and Drug Administration for clinical use in MM and, in the last 5 years, a lot of clinical and preclinical research has been done to optimize the use of this drug class. Currently, mAbs have already become part of standard-of-care combinations for the treatment of relapsed/refractory MM and very soon they will also be used in the frontline setting. The success of simple mAbs (‘naked mAbs’) prompted the development of new types of molecules. Antibody–drug conjugates (ADCs) are tumor-targeting mAbs that release a cytotoxic payload into the tumor cells upon antigen binding in order to destroy them. Bispecific antibodies (BiAbs) are mAbs simultaneously targeting a tumor-associated antigen and an immune cell-associated antigen in order to redirect the immune cell cytotoxicity against the tumor cell. These different constructs produced solid preclinical data and promising clinical data in phase I/II trials. The aim of this review article is to summarize all the recent developments in the field, including data on naked mAbs, ADCs and BiAbs.

## 1. Introduction

Multiple myeloma (MM) is the second most common hematologic malignancy, with approximately 5:100,000 new cases per year in Western countries [1]. Although the introduction of new pharmacologic classes, such as proteasome inhibitors (PIs) or immunomodulatory drugs (IMiDs), has revolutionized treatment in the last decades [2], MM remains an incurable disease and almost all patients relapse after a variable period and become refractory to previously used drugs (relapsed/refractory multiple myeloma, RRMM). In clinical practice, the development and introduction of new drugs with unique mechanisms of action and the combination of different drug classes have increased treatment options for RRMM patients and have improved the depth of response in newly diagnosed (ND) MM patients [3].

**Figure 1:**
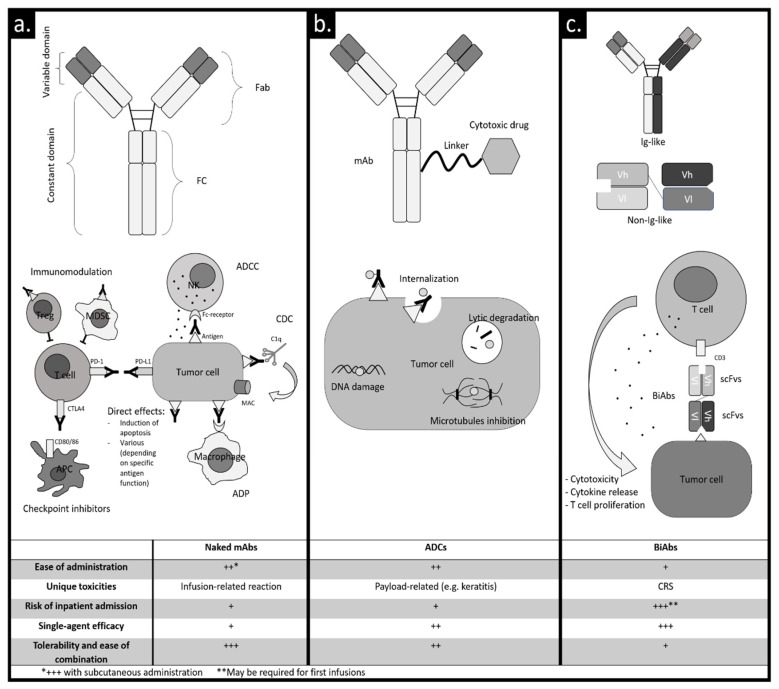
Monoclonal antibodies: types and mechanisms of action. (**a**) Naked monoclonal antibody (mAb). Antibody-dependent cellular cytotoxicity (ADCC): natural killer cell (NK) binds to the Fc region via the Fc-receptor and releases lytic factors such as perforin and granzymes. Complement-dependent cytotoxicity (CDC): interaction between the Fc region and protein C1q activates the classic complement pathway that results in the formation of membrane attack complex (MAC) and cell lysis. Antibody-dependent phagocytosis (ADP): the binding of macrophages induces activation of phagocytosis. Direct effects: induction of apoptosis directly or through cross-linking; effects depending on specific antigen functions (e.g., inhibition of intracellular signaling, blocking of enzymatic functions). Checkpoint inhibitors: blockade of programmed cell death protein 1 (PD-1) or cytotoxic T-lymphocyte antigen 4 (CTLA-4) preventing immune response suppression. Immunomodulation: interaction of mAb with stroma cells, which inhibit T cell activation restoring the immune response against neoplastic cells. (**b**) Antibody–drug conjugate. Upon antibody binding to the target, a cytotoxic payload is released in the target cell. (**c**) Bispecific monoclonal antibodies. Ig-like: two binding sites with different specificity and an Fc region that binds to the Fc-receptor. Non-Ig-like: two different single-chain variable fragments (variable regions comprised only of the variable regions of the heavy and light chains). Bispecific monoclonal antibodies usually bind a tumor antigen and an immune effector antigen (e.g., CD3 on the T-cell surface), in order to activate the immune cells against the neoplastic cell. *Abbreviations*: +/++/+++, low/moderate/high; Fab, fragment antigen binding; FC, fragment crystallizable region; Treg, regulatory T cell; MDSC, myeloid-derived suppressor cell; NK, natural killer cell; ADCC, antibody-dependent cellular cytotoxicity; CDC, complement-dependent cytotoxicity; MAC, membrane attack complex; ADP, antibody-dependent phagocytosis; APC, antigen-presenting cell; ADCs, antibody–drug conjugates; BiAbs, bispecific antibodies; CTLA-4, cytotoxic T-lymphocyte antigen 4; PD-1, programmed cell death protein 1; PD-L1, programmed death ligand 1; di-scFv, bivalent single-chain variable fragment; scFv, single-chain variable fragment; CRS: cytokine release syndrome; Ig, immunoglobulin.

In MM, the dysregulation of the immune system plays an important role in the development and progression of the disease. Producing drugs acting on the complex interplay between the immune system and the tumor cells (i.e., immunotherapy) is thus an appealing strategy [4,5]. There are different types of immunotherapy [6]. Cellular immunotherapies usually involve the harvesting of various immune cell populations (e.g., T lymphocytes and natural killer cells) that are properly stimulated or genetically modified ex vivo and then infused into the patients to target tumor cells [7]. A simpler form of immunotherapy relies on the infusion of target-specific antibodies produced from a single clone (monoclonal antibodies, mAbs) that target neoplastic cells and activate the immune system or disrupt a signaling pathway protecting neoplastic cells from immune-cell destruction. In this review, we will focus on mAb-related immunotherapies (Figure 1).

In 1975, Köhler and Milstein developed a technology to produce specific clonal antibodies targeting a desired antigen (mAbs) by fusing mouse MM cell lines with spleen cells from an immunized donor [8]. Starting from this pivotal study, we are now able to produce chimeric, humanized and also totally human mAbs for clinical use.

MAbs without added elements are called “naked” and are composed of two fragment antigen-binding regions (Fab) and a fragment crystallizable region (Fc). The two Fab are responsible for the interaction with the antigen. The Fc is responsible for the interaction with cells that express an Fc receptor (lymphocytes, macrophages, neutrophils and dendritic cells) and with some proteins of the complement system [9] (Figure 1a). 

Fc mediates three main mechanisms of immune activation: antibody-dependent cellular cytotoxicity (ADCC) in which effector cells, typically NK cells [10], are activated and the release of lytic enzymes leads to cytotoxic effects; complement-dependent cytotoxicity (CDC) in which the binding of protein C1q triggers the activation of the classical complement pathway [11]; antibody-dependent phagocytosis (ADP) in which macrophages are activated to phagocytize neoplastic cells [12]. Furthermore, the Fc receptor may induce programmed cell death through the cross-linking of some target antigens [13]. Besides Fc-dependent processes, some mAbs can modulate the enzymatic function of the target cell, ultimately contributing to antineoplastic effects [14]. Naked mAbs can modulate the immune response against tumor cells also without a direct binding to the tumor cell. For instance, the blockade of inhibitory molecules (e.g., PD-1/PD-L1 axis) that usually turn off the immune response can restore the tumor-specific immune function [15,16]. Moreover, the selective targeting of suppressive cells inhibiting the immune response in the tumor microenvironment (e.g., regulatory T cells (Tregs) and myeloid suppressor-derived cells) can restore the immune function as well [17,18].

The structure of naked mAbs can be modified to obtain new mechanisms of action. A naked mAb can be conjugated through a linker to a cytotoxic drug (payload) that is released upon antigen binding into a target cell. In this way, the specific binding of mAbs to the target cell is used to convey a payload directly to the tumor cell. This type of molecule is called an antibody–drug conjugate (ADC) [19] (Figure 1b). Payloads used in ADCs are highly active drugs, usually directed against microtubules or DNA. The binding of the ADC to the antigen induces the internalization of the ADC–antigen complex and the release of the payload via linker cleavage or degradation into lysosomes, reducing systemic adverse effects and achieving a powerful antineoplastic effect. Moreover, the Fc region of the ADC can activate Fc-dependent effects (CDC, ADCC and ADP), contributing to antitumor activity [20].

Recently, mAbs with double specificity (bispecific antibodies, BiAbs, Figure 1c) have been developed. Typically, targeted antigens are a tumor antigen and a molecule expressed on the immune cell surface (for example CD3 on T-lymphocytes), in order to redirect the immune response against tumor cells. BiAbs promote T cell binding to the tumor cell, activation and tumor cell lysis through the direct stimulation of CD3 and thus bypassing the T cell receptor and antigen presentation. Moreover, T cell dependence on antigen presenting cells costimulation and cytokines production is overcome by reducing the risk of T cell anergy. BiAbs can be classified into two categories according to their similarity to the structure of a normal immunoglobulin (Ig): Ig-like and non-Ig-like [21]. Antibodies in the first class have a structure similar to an Ig, they have an Fc region, and the high molecular weight confers a long half-life because they are not directly excreted by the kidney at the cost of poor tissue penetration. Non-Ig-like molecules do not have an Fc region and they are usually small molecules with a high tissue penetration at the cost of a short half-life because they are directly excreted by the kidney. This issue usually requires a continuous infusion in order to obtain clinically active levels of circulating non-Ig-like BiAbs.

Bispecific T-cell engagers (BiTEs) belong to the non-Ig-like group and they are the first BiAbs to have received regulatory approval for the clinical treatment of hematologic malignancies (blinatumomab in acute lymphoblastic leukemia) [22]. BiTEs are composed of two single-chain variable fragments (scFvs, which are small proteins containing only variable regions acting as binding sites) connected with a peptide linker [23]. The first scFv is directed against a tumor antigen, and the other one against CD3 expressed by T cells. BiTEs colocalize T cells and tumor cells and activate T cells against the tumor cells independently of T cell receptor specificity (Figure 1c).

In the next sections, clinical data on naked mAbs, ADC and BiAbs will be discussed. The clinical results from key trials including mAbs for the treatment of MM are summarized in Table 1 (phase I-II trials) and Table 2 (phase III trials).

## 2. Naked Monoclonal Antibodies

The first mAb introduced in clinical practice for the treatment of MM was daratumumab, a fully human IgG antibody. Its target is CD38, a multifunctional ectoenzyme with a role in NAD+ metabolism that acts as a receptor [58]. Its high expression on MM cells, Tregs, B reg cells, and myeloid-derived suppressor cells (MDSCs) makes it an ideal antigen to synergistically target MM and revert the immunosuppressive tumor microenvironment [18]. The role of daratumumab in the treatment of MM is well established, both in RRMM patients and, recently, also in NDMM patients. Its efficacy and safety encouraged the development of other mAbs targeting CD38.

Daratumumab was first approved in 2015 by the US Food and Drug Administration (FDA) and in 2016 by the European Medicines Agency (EMA), on the basis of phase I/II studies GEN501 and SIRIUS [24,25] in RRMM patients who previously received PIs and IMiDs. The most common toxicity was infusion-related reaction (IRR, 48%), especially with the first dose and mostly of grade (G) 1–2. The combined analysis of the two trials showed an overall response rate (ORR) of 30.4%, with a median overall survival (OS) of 20.5 months [59].

More important results were obtained by combining daratumumab with other drug classes such as IMiDs and PIs. Combination therapies with bortezomib-dexamethasone (Dara-Vd) and lenalidomide-dexamethasone (Dara-Rd) were evaluated in two phase III studies in RRMM patients: CASTOR and POLLUX [48,49]. These two studies led to FDA approval in 2016 and EMA approval in 2017 of daratumumab triplets in RRMM patients.

In the CASTOR study, Dara-Vd and Vd were compared. The primary endpoint was progression-free survival (PFS). With a median follow-up of 19.4 months, PFS was 16.7 months in the Dara-Vd arm vs. 7.1 months in the Vd arm [60]. The most common G3-4 adverse events (AEs) were thrombocytopenia (45.7 vs. 32.9) and peripheral neuropathy (4.5 vs. 6.8). Discontinuation of treatment due to AEs was similar (9.5% vs. 9.3%).

In the POLLUX trial, 569 patients treated with a median of 1 prior line of therapy were randomized to Dara-Rd vs. Rd. With a median follow-up of 44.3 months, PFS was 44.5 months in the Dara-Rd arm vs. 17.5 months in the Rd arm. Moreover, the addition of daratumumab to Rd led to an ORR of 92.9% vs. 76.4% in the control arm, with a minimal residual disease (MRD) negativity rate of 30.4% vs. 5.3%. The most common G3-4 AEs were fatigue, diarrhea, thrombocytopenia, neutropenia and lymphopenia [61].

The third-generation IMiD pomalidomide showed the in vitro ability to upregulate CD38 expression on MM cells and to induce a potential synergistic immunomodulatory effect [62]. Therefore, the association of daratumumab with pomalidomide and dexamethasone (Dara-Pd) was investigated in the phase I/II trial EQUULEUS [26]. In this study, Dara-Pd was administered to 103 heavily pretreated RRMM patients (median of prior lines was 4), obtaining an ORR of 60%, with an MRD negativity rate of 6%. Most common AEs included neutropenia (80%), anemia (50%), fatigue (52%), diarrhea (43%) and thrombocytopenia (42%). The most common hematologic G3-4 toxicity was neutropenia (77%), other G3-4 AEs were comparable to those observed with Pd alone [63]. A phase III trial (APOLLO) evaluated the efficacy of Dara-PD vs. Pd, and recently a press release announced that the primary endpoint of this trial (PFS) was met [64].

Results from the combination of daratumumab with the second-generation PI carfilzomib in the phase III trial CANDOR were recently presented [50]. In this trial, 466 RRMM patients were randomized to receive daratumumab plus carfilzomib and dexamethasone (Dara-Kd) or Kd alone. The ORR was 84% vs. 75%, MRD negativity rate 14% vs. 3%, and PFS not reached (NR) vs. 15.8 months in the Dara-Kd vs. Kd arm respectively. The most frequent G ≥ 3 AEs were thrombocytopenia (24% vs. 16%), respiratory tract infection (29% vs. 16%) and hypertension (18% vs. 13%).

The very good safety profile combined with the synergistic effect with standard-of-care (SOC) combinations in RRMM patients led to exploring the addition of daratumumab to SOC combinations in NDMM patients as well, both in transplant-eligible (TE) and non-transplant-eligible (NTE) patients.

In the phase III trial ALCYONE, 706 NDMM NTE patients were randomized to receive 9 cycles of bortezomib, melphalan and prednisone (VMP) vs. VMP plus daratumumab (Dara-VMP) followed by daratumumab until progression [51]. With a median follow-up of 40.1 months, median PFS was 36.4 vs. 19.3 months, ORR 90.9% vs. 73.9% and the MRD negativity rate 28% vs. 7% in the experimental vs. control arm. A survival advantage of Dara-VMP vs. VMP was also observed (HR 0.60, *p* = 0.0003). AEs were comparable in both arms, with the exception of G3-4 infections (23.1 vs. 14.7% in the daratumumab vs. control arm). 

Another important SOC for NDMM NTE patients is Rd. In the phase III MAIA trial, 737 NDMM NTE patients received Rd with or without daratumumab [52]. With a median follow-up of 28 months, median PFS was NR in the daratumumab group vs. 31.9 months in the Rd group. ORR rates were 92.9% vs. 81.3%, respectively, with a MRD negativity rate of 24.2% vs. 7.3%, thus confirming the greater depth of response achievable in the daratumumab arm. The most common toxicities observed with daratumumab were neutropenia (50% vs. 35.3% in the daratumumab vs. control arm) and pneumonia (13.7% vs. 7.9%), with a low rate of treatment withdrawal in both arms (0.5% vs. 1.4%).

For TE patients, different induction regimens are considered SOC in different countries. The triplet bortezomib, thalidomide and dexamethasone (VTD) is still one of the most used in Europe. In the phase III trial CASSIOPEIA, the efficacy of daratumumab in combination with VTD (Dara-VTD) was evaluated in 1085 NDMM TE patients. The primary endpoint of the study was the rate of the stringent complete response (sCR) 100 days after transplant [53]. This rate was 20% in the VTD arm vs. 29% in the Dara-VTD arm, translating into a significant benefit in PFS (HR 0.47, *p* < 0.0001). The second phase of CASSIOPEIA is ongoing and is evaluating the role of daratumumab in maintenance therapy.

GRIFFIN is another important phase II trial evaluating the efficacy and safety of the addition of daratumumab to the combination of bortezomib, lenalidomide and dexamethasone (Dara-VRd) in NDMM TE patients [27]. With a median follow-up of 22.1 months, the rate of sCR was 62.2% in the daratumumab arm vs. 45.4% in the control arm, with a 24-month PFS of 95.8% vs. 89.8%, respectively. Although there were more cases of neutropenia (41.4% vs. 21.6%), thrombocytopenia (16.2% vs. 8.8%) and infections (90.9% vs. 61.8%) in the daratumumab group, the rate of treatment discontinuation was 15.2% in the Dara-VRd arm vs. 20.6% in the VRd arm. The ongoing phase III trial PERSEUS is evaluating the efficacy of daratumumab combined with VRd in the induction and consolidation phases and with lenalidomide in the maintenance phase [65].

The route of administration of daratumumab is intravenous (Dara iv), with IRRs occurring in about half of the treated patients. The median duration of infusion is about 7 h for the first infusion and 4 h for subsequent infusions [66], with a negative impact on the quality of life of patients, since they need to spend a lot of time receiving infusions in hospital facilities [67]. In the phase Ib trial PAVO, encouraging results were obtained with the subcutaneous administration of daratumumab (Dara sc). These findings were confirmed by the phase III study COLUMBA [28,54]. The sc route of administration was not inferior to the iv route in terms of efficacy (ORR 41% vs. 37%, respectively), while safety was better with the sc route in terms of IRRs (13% vs. 34%). In the PLEIADES study, Dara sc added to SOC regimens showed a clinical activity similar to that of Dara iv-containing regimens, thus further confirming the superiority of the sc route also in combination regimens [68].

Isatuximab is a chimeric naked mAb targeting CD38 that showed an anti-MM activity similar to that of daratumumab, with a peculiar proapoptotic effect [69]. Phase I studies showed its efficacy both as a single agent and in combination therapy [29,30,70].

Isatuximab was recently approved by FDA and EMA in combination with Pd, based on the results of the phase III ICARIA-MM trial. In this study, 307 RRMM patients with a median of 3 prior lines of therapy were enrolled to receive Pd with or without isatuximab [55]. The ORR was 60% vs. 35% in the isatuximab vs. the control arm. With a median follow-up of 11.6 months, PFS was 11.5 vs. 6.5 months in the isatuximab vs. the control group. As with daratumumab, IRRs with isatuximab were common, mostly observed during the first infusion and rarely severe (3% of IRRs were G3-4).

Another phase III trial enrolled 302 RRMM patients to compare isatuximab-Kd to Kd alone [56]. After a median follow-up of 20.7, PFS was significantly better in the experimental vs. the control arm (HR 0.531, *p* = 0.0007). The MRD negativity rate was higher in the experimental arm as well (29.6% vs. 13.0%). G ≥ 3 respiratory infections were observed in 32.2% of patients in the isatuximab-Kd arm vs. 23.8% in the Kd arm. 

The phase III IMROZ trial will evaluate the efficacy of isatuximab in combination with VRD in NDMM NTE patients [71]. In the phase Ib GMMC-CONCEPT trial, isatuximab was evaluated in combination with the triplet KRD in high-risk NDMM patients. First data were recently presented at the ASCO 2020 Annual Meeting, showing an encouraging ORR of 100% [31].

Other two anti-CD38 mAbs, MOR202 and TAK-079, are under investigation. MOR202 is an iv anti-CD38 mAb without CDC activity. Clinically, it showed a lower frequency of IRRs compared to Dara iv. However, the ORR of MOR202 in combination with dexamethasone, lenalidomide or pomalidomide (28%, 65% or 43% respectively) was not higher than that of other anti-CD38 mAbs, despite the limitations of cross-trial comparisons [32].

Recent data on TAK-079 (mezagitamab) showed an ORR of 33% at the dose of 600 mg sc in heavily pretreated RRMM patients (4 median prior lines of therapy, including patients exposed to other anti-CD38 mAbs). Its advantages are the sc route of administration and a promising safety profile (no IRRs, no significant hematologic toxicity) [33].

Elotuzumab is another naked mAb used in clinical practice. Its molecular target is the surface glycoprotein signaling lymphocytic activation molecule family 7 (SLAMF7), which is mainly expressed by NK and normal or neoplastic plasma cells, promoting their growth and survival and mediating their interaction with the microenvironment. Therefore, the elotuzumab mechanism of action involves a blockade of tumor interactions [72], growth and survival signals. Moreover, it stimulates NK cells by enhancing their ADCC activity [73,74]. In phase I studies, elotuzumab showed no efficacy as a single agent [75], but encouraging in vitro studies found a potential synergistic effect in combination with IMiDs [76,77]. This synergy was clinically evaluated in the ELOQUENT-2 and ELOQUENT-3 trials by adding elotuzumab to Rd and Pd. Positive results from both trials led to the approval of these combinations in RRMM patients [34,57].

In NDMM patients, instead, elotuzumab did not show encouraging results. The ELOQUENT-1 trial evaluated the triplet elotuzumab-Rd in NTE NDMM patients, and it was recently announced that the primary endpoint (PFS) was not met [78].

An interesting characteristic of elotuzumab is its safety profile, with the low rate of IRRs and the absence of additional toxicity making it a good option for the treatment of frail patients.

Immune checkpoints shutting down the antineoplastic immune response are important molecules involved in tumorigenesis, and the PD-1/PD-L1 axis is one of the most important pathways working as an immune checkpoint. Many naked mAbs interact with it, blocking PD-1 (nivolumab, pembrolizumab and cepilimab) or PD-L1 (durvalumab and atezolizumab). The importance of these mAbs in cancer immunotherapy is well known, and MM cells and their microenvironment seem to rely on the PD-1/PD-L1 interaction, thus fostering the design of clinical studies with immune checkpoint inhibitors in MM.

Pembrolizumab as a single agent did not show efficacy [79], while, in phase I studies, its combination with lenalidomide and pomalidomide showed an ORR of 44% and 60%, respectively [35,36]. Unfortunately, both the KEYNOTE-183 trial (RRMM patients) and the KEYNOTE-185 trial (NDMM NTE patients) were discontinued due to severe toxicities [80,81]. Although these results reduced the interest in checkpoint inhibitors in MM, there are encouraging preclinical data about their use in combination with other mAbs: it seems indeed that the combination with elotuzumab may increase NK peritumoral infiltration and cytokine release [82]. Moreover, the combination with daratumumab showed a synergistic anti-MM effect [83].

B-cell maturation antigen (BCMA) is another very specific antigen expressed almost exclusively by plasma cells. This surface protein is an interesting target because it promotes survival and growth when interacting with its ligands, BAFF and APRIL [84]. The first naked anti-BCMA mAb was cSG1 [85], which showed anti-MM effects, but was not further developed. A phase I trial exploring a humanized, non-fucosylated IgG1 anti-BCMA naked mAb is ongoing [86]. Nonetheless, due to its specificity, BCMA is the ideal target for more powerful immunotherapies such as ADCs and BiAbs, as will be discussed in the next section. 

## 3. ADCs

ADCs are a rapidly growing class of immunotherapeutic agents. Different constructs, payloads and target antigens are in preclinical or early clinical investigation for the treatment of MM [87]. Among them, the most promising agent of which we already have clinical data is belantamab mafodotin (belamaf), a humanized anti-BCMA IgG mAb fused to the payload monomethyl auristatin F (MMAF). In preclinical in vitro and in vivo models, belamaf showed anti-MM activity without affecting BCMA-negative cells and the MMAF arrested the cell cycle of malignant plasma cells at the G2/M phase, eventually leading to cell death [88]. Its afucosylated Fc fraction promotes Fc-dependent immune effector functions, mainly ADCP and ADCC [89].

In a first-in-human phase I trial in heavily pretreated RRMM, single-agent belamaf was tested at different dose levels. The dose level of 3.4 mg/Kg given every 21 days was further tested and expanded to 35 patients. At this dose level, the drug was associated with an ORR of 60% and a PFS of 12 months [38]. IRRs were mild and infrequent (29%, mostly G1-2). The 2 main emerging toxicities were thrombocytopenia (63%, 35% of which G ≥ 3) and keratopathy (69% of patients, 14% of which G ≥ 3). Keratopathy is a well-known side effect of MMAF and, though the exact mechanism of toxicity is unknown, it may be due to a non-specific and BCMA-independent uptake of the drug in the basal epithelial layer of the cornea [90]. From a practical standpoint, belamaf infusion lasts 30 min and no inpatient admission is required.

Following these encouraging results, a phase II trial designed for patients refractory to a PI, an IMiD, and an anti-CD38 mAb evaluated both the 3.4 and the 2.5 mg/kg dose levels [39]. Safety and efficacy were comparable between the two dose levels. At 2.5 mg/kg, the ORR was 31% and median PFS was 2.9 months. This study confirmed the frequent occurrence of keratopathy detected on eye examination (72%, any grade). However, few patients experienced ocular symptoms (G3-4 in <5% of patients) and, importantly, keratopathy was always reversible [40]. Indeed, although visual acuity was affected in 18% of treated patients, 82% of them recovered at the current follow-up. Two post-hoc analyses of this trial demonstrated that belamaf was active in patients with high-risk cytogenetics and that renal impairment up to 30 mL/min of the estimated glomerular filtration rate did not impact the efficacy and tolerability of this drug [41,42].

A trial evaluating the addition of belamaf to standard MM backbone treatments (Rd and Vd) is ongoing in RRMM (DREAMM-6). Recently, data about the first 18 patients treated with belamaf-Vd were presented [43]. Efficacy was good, with a high ORR (78%). However, as expected in a bortezomib-based combination, thrombocytopenia was frequent and severe (G3 17% and G4 44%).

The development of other ADCs was recently reviewed elsewhere [87]. 

## 4. BiAbs

BCMA on malignant plasma cells and CD3 on T cells are the two main targets exploited to design anti-MM BiAbs. Other BiAbs targeting different antigens on the plasma cell surface and/or involving different immune effectors have been reviewed elsewhere [91].

AMG 420 is an anti-BCMA BiTE that was tested in a dose-escalation first-in-human study enrolling RRMM patients. At the maximum tolerated dose (400 mcg/die), a very good efficacy was reported (ORR 70%) [44]. Due to the pharmacokinetics typical of non-Ig-like BiAbs, this drug formulation required a continuous infusion for 4 weeks on therapy followed by 2 weeks off therapy. Infections were frequent (G ≥ 3 24%), and the use of a central venous catheter line to deliver the drug led to central line infections in 12% of patients. Other treatment-emergent AEs were cytokine release syndrome (CRS, 38%, mostly G1-2) and peripheral neuropathy (G 3 5%). 

Due to the aforementioned pharmacokinetic issues, AMG 420 has not been further developed. Nonetheless, a study evaluating AMG 701, a half-life extended BiTE not needing continuous infusion, is currently ongoing [92].

PF-06863135 (PF-3135) is a humanized Ig-like BiAb that is currently being tested in a dose-escalation study in RRMM [45]. Due to its Ig-like structure, PF-3135 is infused once weekly. Results of the first 17 patients showed a minimal response in 1 patient (6%), although the clinical benefit rate (defined as best response ≥ stable disease) was 41% and dose escalation is still ongoing. Three patients (18%) experienced G ≥ 3 AEs and the only non-hematologic AE was an increase in blood liver enzymes in 1 patient.

CC-93269 is an Ig-like BiAb asymmetrically targeting BCMA through two binding sites and targeting CD3 through one binding site [46]. A dose-escalation phase I study in heavily pretreated RRMM patients is ongoing and results of the first 30 patients have been presented. CC-93269 was administered intravenously over 2 h: weekly in cycles 1–3, every other week in cycles 4–6, and every 28 days thereafter. The ORR was 43% throughout the dose cohorts, but it became dose-dependent, reaching 89% at the highest tested dose (10 mg). CRS was mild but frequent (all grades 77%; G ≥ 3 4%). Thus, dexamethasone prophylaxis was implemented in patients treated with doses >6 mg. The main toxicities were neutropenia (G ≥ 3 43%) and infections (G ≥ 3 30%).

Teclistamab is another Ig-like BiAb. Results of the first 78 RRMM patients enrolled in a phase I dose-escalation trial were recently presented [47]. Priming doses followed by weekly iv infusions were administered at different dose levels (0.3–720 mcg/Kg). CRS was common (56%), but all cases were G1 or 2. Neurotoxicity was reported in 8% of treated patients. An ORR of 67% was observed in the 12 patients treated at the highest dose (270 mcg/Kg), while no efficacy data from the 720 mcg/kg cohort are yet available.

## 5. Future Directions and Conclusions

The introduction of mAbs for the treatment of MM has already changed clinical practice in RRMM patients, leading to better outcomes. Moreover, between 2019 and 2020, daratumumab combinations with Rd and VMP in NTE NDMM patients and with VTd in TE NDMM patients were approved by FDA and EMA. This means that the great majority of NDMM patients will receive an anti-CD38 naked mAb in the near future, due to the higher efficacy of combinations with daratumumab and to its negligible toxicity when added to SOC regimens. In the next years, more and more patients will eventually be exposed or refractory to anti-CD38 naked mAbs after first relapse, thus questioning the current treatment sequencing in RRMM patients. Initial reports of the suboptimal efficacy of retreatment with anti-CD38 mAbs in a small series of patients are beginning to emerge [93] and will require prospective confirmation in a significant number of patients. This issue may be overcome by using different anti-CD38 mAbs with unique mechanisms of action [94]. For example, differently from daratumumab, isatuximab mediates a direct cytotoxic effect against MM cells independently of the presence of Fc-cross-linking agents [69]. MOR202 does not induce CDC, decreasing the IRR rate at the cost of a reduced single-agent activity [32]. TAK-079 minimally binds to targets with a low density of CD38, leading to an enhanced depletion of high-density CD38+ target cells [95]. However, the predicted efficacy of these mAbs is largely dependent on the mechanisms of resistance to anti-CD38 mAbs. Resistance to ADCC (e.g., fratricidal depletion of CD38+ NK cells), CDC (e.g., upregulation of complement-inhibitory molecules) and ADCP (e.g., upregulation of CD47 inhibiting phagocytosis) have been observed during anti-CD38 mAb treatment and strategies to overcome them are under clinical investigation [96]. Nevertheless, the most relevant issue limiting retreatment with anti-CD38 mAbs is the long-lasting downregulation of CD38 on plasma cell surfaces after anti-CD38 therapy [97]. Even though strategies to reinduce CD38 expression in malignant plasma cells are under clinical investigation [98,99], changing the target antigen may be a more appealing strategy in RRMM patients who are refractory to anti-CD38 treatment. The new anti-BCMA molecules could find their therapeutic space in this scenario.

Several observations can be made about the comparison among naked mAbs, ADCs and BiAbs. Single-agent activity of naked mAbs is relatively low (ORR around 20–30% in heavily pretreated RRMM patients with anti-CD38 mAbs), while ADCs (ORR up to 60% in RRMM with belamaf) and BiAbs (ORR up to 90% in RRMM with CC-93269) can induce deeper responses. However, naked mAbs are very safe drugs and do not have overlapping toxicities with other MM drugs. As a consequence, they may be easily combined with MM backbone treatments. Moreover, they may be effortlessly administered in outpatient facilities, and their subcutaneous formulations will be available in the future. ADCs have toxicity profiles non-overlapping with IMiDs and PIs and clinical trials exploring ADC-based combination therapies are ongoing. Moreover, they can be easily infused in outpatient facilities as well, although the off-target toxicity of the payload could be an issue (e.g., eye toxicity with belamaf). BiAbs are very effective drugs, but the infection risk makes it difficult to combine them with other MM backbones. Moreover, the strong activation of the immune system and consequent CRS risk may require preventive hospitalization during the first days after treatment or at least a close monitoring of CRS symptoms.

Other types of anti-BCMA immunotherapy, such as CAR T-cell therapy, are currently available However, despite their high efficacy as single agents (up to an ORR of 100%) [100], they are currently not ‘off-the-shelf’ drugs. Differently from naked mAbs/ADCs/BiAbs, they require specialized centers and inpatient admission for the infusion. Furthermore, CRS/neurotoxicity should be closely monitored and promptly treated.

Currently, the optimal scenario for each of these drugs could depend on both disease risk and patient fitness. For instance, intermediate-fit or frail patients may safely receive naked mAbs or ADCs, but they are unlikely to tolerate BiAbs or CAR T-cell therapy. On the other hand, fit patients who present with high-risk disease or who experienced early relapse after first-line treatment may benefit from BiAbs or CAR T-cell therapy [101]. As with anti-CD38 therapies, the mechanisms of resistance to anti-BCMA agents may shed light on the optimal treatment sequencing. For instance, antigen escape with relapse guided by BCMA-low or BCMA-negative malignant plasma cells has been described during anti-BCMA CAR T-cell treatment, predicting cross-resistance with other anti-BCMA agents [7].

New data from prospective studies will help us understand the best drug to be used in each setting.

In conclusion, therapeutic options in MM are continuously emerging, and mAbs are greatly contributing, and will contribute even more in the future, to improving the outcome of MM patients.

## Figures and Tables

**Table 1:** List of phase I and II trials exploring mAbs in multiple myeloma (MM)

MAb Class	Molecule (Targets)	Study	Treatment	Setting	Toxicities (≥G3)	ORR (MRD Negativity Rate; NGS, Sensitivity 10^−5^)	PFS	OS
Naked	Daratumumab (anti-CD38)	GEN501 + SIRIUS [24,25] NCT00574288 NCT01985126	Daratumumab single agent	RRMM	Anemia (17.6%); back pain g3 (2.7%); fatigue g3 (2%)	31.1%	4	20.1
Naked	Daratumumab (anti-CD38)	EQUULEUS [26] NCT01998971	Dara-Poma-dex	RRMM	Neutropenia (77%); fatigue (12%); dyspnea (8%)	60% (6%)	8.8	17.5
Naked	Daratumumab (anti-CD38)	GRIFFIN [27] NCT02874742	Dara-VRd vs. VRd	NDMM	Neutropenia (41.4% vs. 21.6%); peripheral neuropathy (7.1% vs. 7.8%); diarrhea (7.1% vs. 3.9%)	99% vs. 91.8% (51% vs. 20.4%)	NR vs. NR	NR vs. NR
Naked	Daratumumab (anti-CD38)	PAVO [28] NCT02519452	Subcutaneous administration of daratumumab single agent*	RRMM	Anemia (15.6%); hypertension (8.9%); pneumonia (4.4%); hyponatremia (4.4%); respiratory syncytial virus infection (4.4%); device-related infection (4.4%)	42.2%	NA	NA
Naked	Isatuximab (anti-CD38)	TCD11863 [29] NCT01749969	Isa-Rd	RRMM	Neutropenia (60%); pneumonia (9%); fatigue (7%)	56%	8.5	NR
Naked	Isatuximab (anti-CD38)	TCD14079 [30] NCT02283775	Isa-Pd	RRMM	Neutropenia (84%); pneumonia (18%); fatigue (7%); urinary tract infection (7%); traumatic fracture (7%); syncope (7%); dyspnea (7%); hypertension (7%)	62.2% (0%) *	17.6	NR
Naked	Isatuximab (anti-CD38)	GMMC-CONCEPT [31] NCT03104842	Isa-KRD	High risk NDMM	Neutropenia (34%); hypertension (12%); cardiac failure (4%)	100% (40%) **	NA	NA
Naked	MOR202 (anti-CD38)	MOR202C101 [32] NCT01421186	MOR202+dexamethasone	RRMM	Anemia (39%); hypertension (11%); bronchitis (6%); pneumonia (6%); hyperglycemia (6%)	28%	8.4	NA
			MOR202-Rd		Lymphopenia (59%); hypophosphatemia (12%); hypertension (12%)	65%	NR	NA
			MOR202-Pd		Neutropenia (71%); pneumonia (24%); hypertension (19%)	48%	17.5	NA
Naked	TAK-079 (anti-CD38)	TAK-079–1501 [33] NCT03439280	TAK-079 single agent	RRMM	Neutropenia (5%); parainfluenza virus infection (5%); diverticulitis (5%)	33%	NR	NA
Naked	Elotuzumab (anti-SLAMF7)	ELOQUENT-3 [34] NCT02654132	Elo-Pd vs. Pd	RRMM	Neutropenia (13% vs. 27%); infections (13% vs. 22%); hyperglycemia (8% vs. 7%)	53% vs. 26%	10.3 vs. 4.7	NA
Naked	Pembrolizumab (anti-PD-1)	KEYNOTE-023 [35] NCT02036502	Pembrolizumab-Rd	RRMM	Neutropenia (27.4%); hyperglycemia (6.5%); pneumonia (6.5%); atrial fibrillation (3.2%); insomnia (3.2%)	44%	7.2	NR
Naked	Pembrolizumab (anti-PD-1)	HP-00061522 [36] NCT02289222	Pembrolizumab-Pd	RRMM	Neutropenia (42%); hyperglycemia (21%); fatigue (15%); pneumonia 15%)	60%	17.4	NR
ADC	Belantamab mafodotin (anti-BCMA, monomethyl auristatin F payload)	DREAMM-1 [37,38] NCT02064387	Belamaf single agent	RRMM	Thrombocytopenia (35%); keratopathy (14%); diarrhea (12%)	60% ***	12	NR
ADC	Belantamab mafodotin (anti-BCMA, monomethyl auristatin F payload)	DREAMM-2 [39,40,41,42] NCT03525678	Belamaf single agent (data on the 2.5 mg/kg cohort are shown)	RRMM	Thrombocytopenia (20%); keratopathy (27%); hypercalcemia (7%)	31%	2.9	14.9
ADC	Belantamab mafodotin (anti-BCMA, monomethyl auristatin F payload)	DREAMM-6 [43] NCT03544281	Belamaf-Vd	RRMM	Thrombocytopenia (61%); keratopathy (56%); hypercalcemia (7%)	78%	NA	NA
BiAb	AMG 420 (anti-BCMA/anti-CD3)	1351.1 [44] NCT02514239	AMG 420 single agent	RRMM	Infections (24%) neuropathy (5%) CRS (2%)	70% ***	NA	NA
BiAb	PF-3135 (anti-BCMA/anti-CD3)	C1071001 [45] NCT03269136	PF-3135 single agent	RRMM	Increased liver enzymes (6%) neutropenia (6%), lymphopenia (6%)	0% ***	NA	NA
BiAb	CC-93269 (anti-BCMA/anti-CD3)	CC-93269-MM-001 [46] NCT03486067	CC-93269 single agent	RRMM	Neutropenia (43%), infections (30%), general physical deterioration (10%)	89% *** (78%) **	NA	NA
BiAb	Teclistamab (anti-BCMA/anti-CD3)	CR108206 [47] NCT03145181	Teclistamab single agent	RRMM	Neutropenia (48%), infections (21%), neurotoxicity (3%)	67% ***	NA	NA

* Sensitivity 10^−6^; ** Flow. *** At the maximum tolerated dose (MTD) or at the highest dose tested when the MTD has not yet been reached. *Abbreviations*: MAb, monoclonal antibody; G, grade; ORR, overall response rate; CRS, cytokine release syndrome; MRD, minimal residual disease; NGS, next-generation sequencing; PFS, progression-free survival; OS, overall survival; RRMM, relapsed/refractory multiple myeloma; NDMM, newly diagnosed multiple myeloma; Dara, daratumumab; P, Poma, pomalidomide; d, dex, dexamethasone; V, bortezomib; R, lenalidomide; NR, not reached; NA, not available; Isa, isatuximab; SLAMF7, signaling lymphocytic activation molecule family 7; Elo, elotuzumab; PD-1, programmed cell death protein 1; BCMA, B-cell maturation antigen; belamaf, belantamab mafodotin; ADC, antibody–drug conjugate; BiAb, bispecific antibody.

**Table 2:** List of phase III trials exploring naked mAbs in multiple myeloma (MM)

Molecule (Target)	Study	Treatment Schema	Setting	Toxicities (≥G3)	ORR (MRD Negativity Rate, NGS, Sensitivity 10^−5^)	PFS	OS
**Daratumumab (anti-CD38)**	CASTOR [48] NCT02136134	Dara-Vd vs. Vd	RRMM	Thrombocytopenia (45.7% vs. 32.9%); pneumonia (9.9% vs. 10.1%); hypertension (6.6% vs. 0.8%)	83.8% vs. 63.2% (11.6% vs. 2.4%)	16.7 vs. 7.1	NA
**Daratumumab (anti-CD38)**	POLLUX [49] NCT02076009	Dara-Rd vs. Rd	RRMM	Neutropenia (55.5% vs. 41.6%); pneumonia (15.2% vs. 10%); diarrhea (9.9% vs. 3.9%);	92.9% vs. 76.4% (30.4% vs. 5.3%)	44.5 vs. 17.5	1-year OS 92.1% vs. 86.8%
**Daratumumab (anti-CD38)**	CANDOR [50] NCT03158688	Dara-Kd vs. Kd	RRMM	Thrombocytopenia (24% vs. 16%); respiratory tract infection (29% vs. 16%); hypertension (18% vs. 13%)	84% vs. 75% (14% vs. 3%)	NR vs. 15.8	NR vs. NR
**Daratumumab (anti-CD38)**	ALCYONE [51] NCT02195479	Dara-VMP vs. VMP	NDMM	Neutropenia (39.9% vs. 38.7%); infections (23.1% vs. 14.7%); any infusion-related reaction (4.9% vs. na)	90.9% vs. 73.9% (22.3% vs. 6.2%)	NR vs. 18.1	36-month rate: 78% vs. 67.9%
**Daratumumab (anti-CD38)**	MAIA [52] NCT02252172	Dara-Rd vs. Rd	NDMM	Neutropenia (50% vs. 35.3%); infections (32.1% vs. 23.3%); fatigue (8% vs. 3.8%)	92.9% vs. 81.3% (24.2% vs. 7.3%)	NR vs. 31.9	NA
**Daratumumab (anti-CD38)**	CASSIOPEIA [53] NCT02541383	Dara-VTd vs. VTd	NDMM	Neutropenia (28% vs. 15%); stomatitis (13% vs. 16%); peripheral sensory neuropathy (9% vs. 9%)	92.6% vs. 89.9% (64% vs. 44%) *	NA	NA
**Daratumumab (anti-CD38)**	COLUMBA [54] NCT03277105	Subcutaneous vs. intravenous administration of daratumumab	RRMM	Thrombocytopenia (14% vs. 13%); hypertension (3% vs. 6%); febrile neutropenia (2% vs. 3%); back pain (2% vs. 3%)	41% vs. 37%	5.6 vs. 6.1	NA
**Isatuximab (anti-CD38)**	ICARIA-MM [55] NCT02990338	Isa-Pd vs. Pd	RRMM	Neutropenia (85% vs. 70%); pneumonia (16% vs. 14%); dyspnea (4% vs. 1%)	60% vs. 35% (5% vs. 0%)	11.5 vs. 6.5	NA
**Isatuximab (anti-CD38)**	IKEMA [56] NCT03275285	Isa-Kd vs. Kd	RRMM	Respiratory infections (32.2% vs. 23.8%); cardiac failure (4% vs. 4.1%); thrombocytopenia (29.9% vs. 23.8%); neutropenia (19.2% vs. 7.4%) kd, respectively.	86.6% vs. 82.9% (29.6% vs. 13%)	NR vs. 19.2	NA
**Elotuzumab (anti-SLAMF7)**	ELOQUENT-2 [57] NCT01239797	Elo-Rd vs. Rd	RRMM	Lymphocytopenia (79% vs. 49%); infections (33% vs. 26%); pneumonia (14% vs. 10%)	79% vs. 66%	19.4 vs. 14.9	48 vs. 40

* Flow. *Abbreviations*: G, grade; ORR, overall response rate; MRD, minimal residual disease; NGS, next-generation sequencing; PFS, progression-free survival; OS, overall survival; RRMM, relapsed/refractory multiple myeloma; NDMM, newly diagnosed multiple myeloma; Dara, daratumumab; d, dex, dexamethasone; V, bortezomib; R, lenalidomide; K, carfilzomib; VMP, bortezomib-melphalan-prednisone; T, thalidomide; P, Poma, pomalidomide; SLAMF7, signaling lymphocytic activation molecule family 7; Elo, elotuzumab; NR, not reached; NA, not available.

## Abbreviations

ADC	antibody–drug conjugate
ADCC	antibody-dependent cellular cytotoxicity
ADP	antibody-dependent phagocytosis
APC	antigen-presenting cell
BCMA	B-cell maturation antigen
belamaf	belantamab mafodotin
BiAb	bispecific antibody
BiTE	bispecific T-cell engager
CDC	complement-dependent cytotoxicity
CRS	cytokine release syndrome
CTLA-4	cytotoxic T-lymphocyte antigen 4
d, dex	dexamethasone
Dara	daratumumab
di-scFv	bivalent single-chain variable fragment
Elo	elotuzumab
EMA	European Medicines Agency
Fab	fragment antigen binding
Fc	fragment crystallizable region
FDA	US Food and Drug Administration
G	grade
Ig	immunoglobulin
IMiDs	immunomodulatory drugs
IRR	infusion-related reaction
Isa	isatuximab
iv	intravenous
K	carfilzomib
mAb	monoclonal antibody
MAC	membrane attack complex
MDSC	myeloid-derived suppressor cell
MM	multiple myeloma
MMAF	monomethyl auristatin F
MRD	minimal residual disease
NA	not available
NDMM	in newly diagnosed (ND) MM
NK	natural killer cell
NR	not reached
NTE	non-transplant-eligible
ORR	overall response rate
OS	overall survival
P, Poma	pomalidomide
PD-1	programmed cell death protein 1
PD-L1	programmed death ligand 1
PFS	progression-free survival
PI	proteasome inhibitor
R	lenalidomide
RRMM	relapsed/refractory multiple myeloma
sc	subcutaneous
scFv	single-chain variable fragment
sCR	stringent complete response
SLAMF7	signaling lymphocytic activation molecule family 7
T	thalidomide
TE	transplant-eligible
Treg	regulatory T cell
V	bortezomib
VMP	bortezomib-melphalan-prednisone

## References

[1] Kazandjian D. (2016). Multiple myeloma epidemiology and survival: A unique malignancy. Semin. Oncol..

[2] Kumar S.K., Dispenzieri A., Lacy M.Q., Gertz M.A., Buadi F.K., Pandey S., Kapoor P., Dingli D., Hayman S.R., Leung N. (2014). Continued improvement in survival in multiple myeloma: Changes in early mortality and outcomes in older patients. Leukemia.

[3] D’agostino M., Bertamini L., Oliva S., Boccadoro M., Gay F. (2019). Pursuing a curative approach in multiple myeloma: A review of new therapeutic strategies. Cancers.

[4] Gay F., D’Agostino M., Giaccone L., Genuardi M., Festuccia M., Boccadoro M., Bruno B. (2017). Immuno-oncologic Approaches: CAR-T Cells and Checkpoint Inhibitors. Clin. Lymphoma Myeloma Leuk..

[5] D’Agostino M., Boccadoro M., Smith E.L. (2017). Novel Immunotherapies for Multiple Myeloma. Curr. Hematol. Malig. Rep..

[6] Galluzzi L., Vacchelli E., Bravo-San Pedro J.M., Buqué A., Senovilla L., Baracco E.E., Bloy N., Castoldi F., Abastado J.P., Agostinis P. (2014). Classification of current anticancer immunotherapies. Oncotarget.

[7] D’Agostino M., Raje N. (2020). Anti-BCMA CAR T-cell therapy in multiple myeloma: Can we do better?. Leukemia.

[8] Köhler G., Milstein C. (1975). Continuous cultures of fused cells secreting antibody of predefined specificity. Nature.

[9] Morgan B.P. (1999). Regulation of the complement membrane attack pathway. Crit. Rev. Immunol..

[10] Wang W., Erbe A.K., Hank J.A., Morris Z.S., Sondel P.M. (2015). NK cell-mediated antibody-dependent cellular cytotoxicity in cancer immunotherapy. Front. Immunol..

[11] Meyer S., Leusen J.H.W., Boross P. (2014). Regulation of complement and modulation of its activity in monoclonal antibody therapy of cancer. MAbs.

[12] Gül N., Babes L., Siegmund K., Korthouwer R., Bögels M., Braster R., Vidarsson G., Ten Hagen T.L.M., Kubes P., Van Egmond M. (2014). Macrophages eliminate circulating tumor cells after monoclonal antibody therapy. J. Clin. Investig..

[13] Overdijk M.B., Jansen J.H.M., Nederend M., Lammerts van Bueren J.J., Groen R.W.J., Parren P.W.H.I., Leusen J.H.W., Boross P. (2016). The Therapeutic CD38 Monoclonal Antibody Daratumumab Induces Programmed Cell Death via Fcγ Receptor–Mediated Cross-Linking. J. Immunol..

[14] Lammerts van Bueren J., Jakobs D., Kaldenhoven N., Roza M., Hiddingh S., Meesters J., Voorhorst M., Gresnigt E., Wiegman L., Ortiz Buijsse A. (2014). Direct In Vitro Comparison of Daratumumab with Surrogate Analogs of CD38 Antibodies MOR03087, SAR650984 and Ab79. Blood.

[15] Oliva S., Troia R., D’Agostino M., Boccadoro M., Gay F. (2018). Promises and pitfalls in the use of PD-1/PD-L1 inhibitors in multiple myeloma. Front. Immunol..

[16] D’Agostino M., Gazzera G., Cetani G., Bringhen S., Boccadoro M., Gay F. (2019). Clinical and pharmacologic features of monoclonal antibodies and checkpoint blockade therapy in multiple myeloma. Curr. Med. Chem..

[17] Bonello F., D’Agostino M., Moscvin M., Cerrato C., Boccadoro M., Gay F. (2018). CD38 as an immunotherapeutic target in multiple myeloma. Expert Opin. Biol..

[18] Krejcik J., Casneuf T., Nijhof I.S., Verbist B., Bald J., Plesner T., Syed K., Liu K., van de Donk N.W.C.J., Weiss B.M. (2016). Daratumumab depletes CD38+ immune regulatory cells, promotes T-cell expansion, and skews T-cell repertoire in multiple myeloma. Blood.

[19] Bouchard H., Viskov C., Garcia-Echeverria C. (2014). Antibody-drug conjugates—A new wave of cancer drugs. Bioorg. Med. Chem. Lett..

[20] Skaletskaya A., Setiady Y.Y., Park P.U., Lutz R.J. (2011). Lorvotuzumab mertansine (IMGN901) immune effector activity and its effect on human NK cells. Cancer Res..

[21] Fan G., Wang Z., Hao M., Li J. (2015). Bispecific antibodies and their applications. J. Hematol. Oncol..

[22] Kantarjian H., Stein A., Gökbuget N., Fielding A.K., Schuh A.C., Ribera J.-M., Wei A., Dombret H., Foà R., Bassan R. (2017). Blinatumomab versus Chemotherapy for Advanced Acute Lymphoblastic Leukemia. N. Engl. J. Med..

[23] Ahmad Z.A., Yeap S.K., Ali A.M., Ho W.Y., Alitheen N.B.M., Hamid M. (2012). ScFv antibody: Principles and clinical application. Clin. Dev. Immunol..

[24] Lokhorst H.M., Plesner T., Laubach J.P., Nahi H., Gimsing P., Hansson M., Minnema M.C., Lassen U., Krejcik J., Palumbo A. (2015). Targeting CD38 with Daratumumab Monotherapy in Multiple Myeloma. N. Engl. J. Med..

[25] Lonial S., Weiss B.M., Usmani S.Z., Singhal S., Chari A., Bahlis N.J., Belch A., Krishnan A., Vescio R.A., Mateos M.V. (2016). Daratumumab monotherapy in patients with treatment-refractory multiple myeloma (SIRIUS): An open-label, randomised, phase 2 trial. Lancet.

[26] Chari A., Suvannasankha A., Fay J.W., Arnulf B., Kaufman J.L., Ifthikharuddin J.J., Weiss B.M., Krishnan A., Lentzsch S., Comenzo R. (2017). Daratumumab plus pomalidomide and dexamethasone in relapsed and/or refractory multiple myeloma. Blood.

[27] Voorhees P.M., Kaufman J.L., Laubach J., Sborov D.W., Reeves B., Rodriguez C., Chari A., Silbermann R., Costa L.J., Anderson L.D. (2020). Daratumumab, lenalidomide, bortezomib, and dexamethasone for transplant-eligible newly diagnosed multiple myeloma: The GRIFFIN trial. Blood.

[28] Usmani S.Z., Nahi H., Mateos M.V., van de Donk N.W.C.J., Chari A., Kaufman J.L., Moreau P., Oriol A., Plesner T., Benboubker L. (2019). Subcutaneous delivery of daratumumab in relapsed or refractory multiple myeloma. Blood.

[29] Martin T., Baz R., Benson D.M., Lendvai N., Wolf J., Munster P., Lesokhin A.M., Wack C., Charpentier E., Campana F. (2017). A phase 1b study of isatuximab plus lenalidomide and dexamethasone for relapsed/refractory multiple myeloma. Blood.

[30] Mikhael J., Richardson P., Usmani S.Z., Raje N., Bensinger W., Karanes C., Campana F., Kanagavel D., Dubin F., Liu Q. (2019). A phase 1b study of isatuximab plus pomalidomide/dexamethasone in relapsed/refractory multiple myeloma. Blood.

[31] Weisel K., Asemissen A.M., Besemer B., Haenel M., Blau I.W., Goerner M., Ko Y.-D., Dürig J., Staib P., Mann C. (2020). Depth of response to isatuximab, carfilzomib, lenalidomide, and dexamethasone (Isa-KRd) in front-line treatment of high-risk multiple myeloma: Interim analysis of the GMMG-CONCEPT trial. J. Clin. Oncol..

[32] Raab M.S., Engelhardt M., Blank A., Goldschmidt H., Agis H., Blau I.W., Einsele H., Ferstl B., Schub N., Röllig C. (2020). MOR202, a novel anti-CD38 monoclonal antibody, in patients with relapsed or refractory multiple myeloma: A first-in-human, multicentre, phase 1–2a trial. Lancet Haematol..

[33] Krishnan A.Y., Patel K.K., Hari P., Jagannath S., Niesvizky R., Silbermann R.W., Berg D.T., Li Q., Allikmets K., Stockerl-Goldstein K. (2020). A phase Ib study of TAK-079, an investigational anti-CD38 monoclonal antibody (mAb) in patients with relapsed/ refractory multiple myeloma (RRMM): Preliminary results. J. Clin. Oncol..

[34] Dimopoulos M.A., Dytfeld D., Grosicki S., Moreau P., Takezako N., Hori M., Leleu X., LeBlanc R., Suzuki K., Raab M.S. (2018). Elotuzumab plus Pomalidomide and Dexamethasone for Multiple Myeloma. N. Engl. J. Med..

[35] Mateos M.V., Orlowski R.Z., Ocio E.M., Rodríguez-Otero P., Reece D., Moreau P., Munshi N., Avigan D.E., Siegel D.S., Ghori R. (2019). Pembrolizumab combined with lenalidomide and low-dose dexamethasone for relapsed or refractory multiple myeloma: Phase I KEYNOTE-023 study. Br. J. Haematol..

[36] Badros A., Hyjek E., Ma N., Lesokhin A., Dogan A., Rapoport A.P., Kocoglu M., Lederer E., Philip S., Milliron T. (2017). Pembrolizumab, pomalidomide, and low-dose dexamethasone for relapsed/refractory multiple myeloma. Blood.

[37] Trudel S., Lendvai N., Popat R., Voorhees P.M., Reeves B., Libby E.N., Richardson P.G., Anderson L.D., Sutherland H.J., Yong K. (2018). Targeting B-cell maturation antigen with GSK2857916 antibody–drug conjugate in relapsed or refractory multiple myeloma (BMA117159): A dose escalation and expansion phase 1 trial. Lancet Oncol..

[38] Trudel S., Lendvai N., Popat R., Voorhees P.M., Reeves B., Libby E.N., Richardson P.G., Hoos A., Gupta I., Bragulat V. (2019). Antibody–drug conjugate, GSK2857916, in relapsed/refractory multiple myeloma: An update on safety and efficacy from dose expansion phase I study. Blood Cancer J..

[39] Lonial S., Lee H.C., Badros A., Trudel S., Nooka A.K., Chari A., Abdallah A.O., Callander N., Lendvai N., Sborov D. (2020). Belantamab mafodotin for relapsed or refractory multiple myeloma (DREAMM-2): A two-arm, randomised, open-label, phase 2 study. Lancet Oncol..

[40] Lonial S., Lee H.C., Badros A., Trudel S., Nooka A.K., Chari A., Abdallah A.-O.A., Callander N.S., Sborov D.W., Suvannasankha A. (2020). Pivotal DREAMM-2 study: Single-agent belantamab mafodotin (GSK2857916) in patients with relapsed/refractory multiple myeloma (RRMM) refractory to proteasome inhibitors (PIs), immunomodulatory agents, and refractory and/or intolerant to anti-CD38 monoclonal antibodies (mAbs). J. Clin. Oncol..

[41] Cohen A.D., Trudel S., Lonial S., Libby E.N., Lee H.C., Besemer B., Facon T., Nooka A.K., Callander N.S., Chari A. (2020). DREAMM-2: Single-agent belantamab mafodotin (GSK2857916) in patients with relapsed/refractory multiple myeloma (RRMM) and high-risk (HR) cytogenetics. J. Clin. Oncol..

[42] Lee H.C., Cohen A.D., Chari A., Hultcrantz M., Nooka A.K., Callander N.S., Suvannasankha A., Badros A., Libby E.N., Trudel S. (2020). DREAMM-2: Single-agent belantamab mafodotin (GSK2857916) in patients with relapsed/refractory multiple myeloma (RRMM) and renal impairment. J. Clin. Oncol..

[43] Nooka A.K., Stockerl-Goldstein K., Quach H., Forbes A., Mateos M.-V., Khot A., Tan A., Abonour R., Chopra B., Rogers R. (2020). DREAMM-6: Safety and tolerability of belantamab mafodotin in combination with bortezomib/dexamethasone in relapsed/refractory multiple myeloma (RRMM). J. Clin. Oncol..

[44] Topp M.S., Duell J., Zugmaier G., Attal M., Moreau P., Langer C., Krönke J., Facon T., Salnikov A.V., Lesley R. (2020). Anti-B-Cell Maturation Antigen BiTE Molecule AMG 420 Induces Responses in Multiple Myeloma. J. Clin. Oncol..

[45] Raje N.S., Jakubowiak A., Gasparetto C., Cornell R.F., Krupka H.I., Navarro D., Forgie A.J., Udata C., Basu C., Chou J. (2019). Safety, Clinical Activity, Pharmacokinetics, and Pharmacodynamics from a Phase I Study of PF-06863135, a B-Cell Maturation Antigen (BCMA)-CD3 Bispecific Antibody, in Patients with Relapsed/Refractory Multiple Myeloma (RRMM). Blood.

[46] Costa L.J., Wong S.W., Bermúdez A., de la Rubia J., Mateos M.V., Ocio E.M., Rodríguez-Otero P., San-Miguel J., Li S., Sarmiento R. (2020). Interim results from the first phase 1 clinical study of the b-cell maturation antigen (BCMA) 2+1 T cell engager (TCE) cc-93269 in patients (PTS) with relapsed/refractory multiple myeloma (RRMM). HemaSphere.

[47] Usmani S.Z., Mateos M.-V., Nahi H., Krishnan A.Y., van de Donk N.W.C.J., San-Miguel J., Oriol A., Rosiñol L., Chari A., Adams H. (2020). Phase I study of teclistamab, a humanized B-cell maturation antigen (BCMA) x CD3 bispecific antibody, in relapsed/refractory multiple myeloma (R/R MM). J. Clin. Oncol..

[48] Palumbo A., Chanan-Khan A., Weisel K., Nooka A.K., Masszi T., Beksac M., Spicka I., Hungria V., Munder M., Mateos M.V. (2016). Daratumumab, Bortezomib, and Dexamethasone for Multiple Myeloma. N. Engl. J. Med..

[49] Dimopoulos M.A., Oriol A., Nahi H., San-Miguel J., Bahlis N.J., Usmani S.Z., Rabin N., Orlowski R.Z., Komarnicki M., Suzuki K. (2016). Daratumumab, Lenalidomide, and Dexamethasone for Multiple Myeloma. N. Engl. J. Med..

[50] Dimopoulos M., Quach H., Mateos M.V., Landgren O., Leleu X., Siegel D., Weisel K., Yang H., Klippel Z., Zahlten-Kumeli A. (2020). Carfilzomib, dexamethasone, and daratumumab versus carfilzomib and dexamethasone for patients with relapsed or refractory multiple myeloma (CANDOR): Results from a randomised, multicentre, open-label, phase 3 study. Lancet.

[51] Mateos M.V., Cavo M., Blade J., Dimopoulos M.A., Suzuki K., Jakubowiak A., Knop S., Doyen C., Lucio P., Nagy Z. (2020). Overall survival with daratumumab, bortezomib, melphalan, and prednisone in newly diagnosed multiple myeloma (ALCYONE): A randomised, open-label, phase 3 trial. Lancet.

[52] Facon T., Kumar S., Plesner T., Orlowski R.Z., Moreau P., Bahlis N., Basu S., Nahi H., Hulin C., Quach H. (2019). Daratumumab plus Lenalidomide and Dexamethasone for Untreated Myeloma. N. Engl. J. Med..

[53] Moreau P., Attal M., Hulin C., Arnulf B., Belhadj K., Benboubker L., Béné M.C., Broijl A., Caillon H., Caillot D. (2019). Bortezomib, thalidomide, and dexamethasone with or without daratumumab before and after autologous stem-cell transplantation for newly diagnosed multiple myeloma (CASSIOPEIA): A randomised, open-label, phase 3 study. Lancet.

[54] Mateos M.V., Nahi H., Legiec W., Grosicki S., Vorobyev V., Spicka I., Hungria V., Korenkova S., Bahlis N., Flogegard M. (2020). Subcutaneous versus intravenous daratumumab in patients with relapsed or refractory multiple myeloma (COLUMBA): A multicentre, open-label, non-inferiority, randomised, phase 3 trial. Lancet Haematol..

[55] Attal M., Richardson P.G., Rajkumar S.V., San-Miguel J., Beksac M., Spicka I., Leleu X., Schjesvold F., Moreau P., Dimopoulos M.A. (2019). Isatuximab plus pomalidomide and low-dose dexamethasone versus pomalidomide and low-dose dexamethasone in patients with relapsed and refractory multiple myeloma (ICARIA-MM): A randomised, multicentre, open-label, phase 3 study. Lancet.

[56] Moreau P., Dimopoulos M.-A., Mikhael J., Yong K., Capra M., Facon T., Hájek R., Spicka I., Risse M.-L., Asset G. (2020). Isatuximab plus carfilzomib and dexamethasone vs carfilzomib and dexamethasone in relapsed/refractory multiple myeloma (ikema): Interim analysis of a phase 3, randomized, open-label study. Proceedings of the EHA25 Virtual Congress.

[57] Lonial S., Dimopoulos M., Palumbo A., White D., Grosicki S., Spicka I., Walter-Croneck A., Moreau P., Mateos M.-V., Magen H. (2015). Elotuzumab Therapy for Relapsed or Refractory Multiple Myeloma. N. Engl. J. Med..

[58] Malavasi F., Deaglio S., Funaro A., Ferrero E., Horenstein A.L., Ortolan E., Vaisitti T., Aydin S. (2008). Evolution and Function of the ADP Ribosyl Cyclase/CD38 Gene Family in Physiology and Pathology. Physiol. Rev..

[59] Usmani S.Z., Weiss B.M., Plesner T., Bahlis N.J., Belch A., Lonial S., Lokhorst H.M., Voorhees P.M., Richardson P.G., Chari A. (2016). Clinical efficacy of daratumumab monotherapy in patients with heavily pretreated relapsed or refractory multiple myeloma. Blood.

[60] Spencer A., Lentzsch S., Weisel K., Avet-Loiseau H., Mark T.M., Spicka I., Masszi T., Lauri B., Levin M.-D., Bosi A. (2018). Daratumumab plus bortezomib and dexamethasone versus bortezomib and dexamethasone in relapsed or refractory multiple myeloma: Updated analysis of CASTOR. Haematologica.

[61] Bahlis N.J., Dimopoulos M.A., White D.J., Benboubker L., Cook G., Leiba M., Ho P.J., Kim K., Takezako N., Moreau P. (2020). Daratumumab plus lenalidomide and dexamethasone in relapsed/refractory multiple myeloma: Extended follow-up of POLLUX, a randomized, open-label, phase 3 study. Leukemia.

[62] Endell J., Boxhammer R., Steidl S. (2014). Synergistic in Vitro Activity of MOR202, a Human CD38 Antibody, in Combination with Pomalidomide. Blood.

[63] San-Miguel J., Weisel K., Moreau P., Lacy M., Song K., Delforge M., Karlin L., Goldschmidt H., Banos A., Oriol A. (2013). Pomalidomide plus low-dose dexamethasone versus high-dose dexamethasone alone for patients with relapsed and refractory multiple myeloma (MM-003): A randomised, open-label, phase 3 trial. Lancet Oncol..

[64] Genmab Genmab Announces European Myeloma Network and Janssen Achieve Positive Topline Results from Phase 3 APOLLO Study of Daratumumab in Combination with Pomalidomide and Dexamethasone in Relapsed or Refractory Multiple Myeloma—Genmab A/S. https://ir.genmab.com/news-releases/news-release-details/genmab-announces-european-myeloma-network-and-janssen-achieve.

[65] Sonneveld P., Broijl A., Gay F., Boccadoro M., Einsele H., Blade J., Dimopoulos M.A., Delforge M., Spencer A., Hajek R. (2019). Bortezomib, lenalidomide, and dexamethasone (VRd) ± daratumumab (DARA) in patients (pts) with transplant-eligible (TE) newly diagnosed multiple myeloma (NDMM): A multicenter, randomized, phase III study (PERSEUS). J. Clin. Oncol..

[66] Janssen Biotech Inc DARZALEX® (Daratumumab) Injection. Full Prescribing Information..

[67] Bittner B., Richter W., Schmidt J. (2018). Subcutaneous Administration of Biotherapeutics: An Overview of Current Challenges and Opportunities. BioDrugs.

[68] Chari A., Rodriguez-Otero P., McCarthy H., Suzuki K., Hungria V., Sureda Balari A., Perrot A., Hulin C., Magen H., Iida S. (2020). Subcutaneous daratumumab plus standard treatment regimens in patients with multiple myeloma across lines of therapy (PLEIADES): An open-label Phase II study. Br. J. Haematol..

[69] Jiang H., Acharya C., An G., Zhong M., Feng X., Wang L., Dasilva N., Song Z., Yang G., Adrian F. (2016). SAR650984 directly induces multiple myeloma cell death via lysosomal-associated and apoptotic pathways, which is further enhanced by pomalidomide. Leukemia.

[70] Martin T., Strickland S., Glenn M., Charpentier E., Guillemin H., Hsu K., Mikhael J. (2019). Phase I trial of isatuximab monotherapy in the treatment of refractory multiple myeloma. Blood Cancer J..

[71] Orlowski R.Z., Goldschmidt H., Cavo M., Martin T.G., Paux G., Oprea C., Facon T. (2018). Phase III (IMROZ) study design: Isatuximab plus bortezomib (V), lenalidomide (R), and dexamethasone (d) vs. VRd in transplant-ineligible patients (pts) with newly diagnosed multiple myeloma (NDMM). J. Clin. Oncol..

[72] Tai Y.-T., Dillon M., Song W., Leiba M., Li X.-F., Burger P., Lee A.I., Podar K., Hideshima T., Rice A.G. (2008). Anti-CS1 humanized monoclonal antibody HuLuc63 inhibits myeloma cell adhesion and induces antibody-dependent cellular cytotoxicity in the bone marrow milieu. Blood.

[73] Pazina T., James A.M., MacFarlane A.W., Bezman N.A., Henning K.A., Bee C., Graziano R.F., Robbins M.D., Cohen A.D., Campbell K.S. (2017). The anti-SLAMF7 antibody elotuzumab mediates NK cell activation through both CD16-dependent and –independent mechanisms. Oncoimmunology.

[74] Collins S.M., Bakan C.E., Swartzel G.D., Hofmeister C.C., Efebera Y.A., Kwon H., Starling G.C., Ciarlariello D., Bhaskar S., Briercheck E.L. (2013). Elotuzumab directly enhances NK cell cytotoxicity against myeloma via CS1 ligation: Evidence for augmented NK cell function complementing ADCC. Cancer Immunol. Immunother..

[75] Zonder J.A., Mohrbacher A.F., Singhal S., van Rhee F., Bensinger W.I., Ding H., Fry J., Afar D.E.H., Singhal A.K. (2012). A phase 1, multicenter, open-label, dose escalation study of elotuzumab in patients with advanced multiple myeloma. Blood.

[76] Lonial S., Vij R., Harousseau J.-L., Facon T., Moreau P., Mazumder A., Kaufman J.L., Leleu X., Tsao L.C., Westland C. (2012). Elotuzumab in combination with lenalidomide and low-dose dexamethasone in relapsed or refractory multiple myeloma. J. Clin. Oncol..

[77] Richardson P.G., Jagannath S., Moreau P., Jakubowiak A.J., Raab M.S., Facon T., Vij R., White D., Reece D.E., Benboubker L. (2015). Elotuzumab in combination with lenalidomide and dexamethasone in patients with relapsed multiple myeloma: Final phase 2 results from the randomised, open-label, phase 1b-2 dose-escalation study. Lancet. Haematol..

[78] Bristol-Myers Squibb Company Press Release Bristol Myers Squibb Reports Primary Results of ELOQUENT-1 Study Evaluating Empliciti (elotuzumab) Plus Revlimid (lenalidomide) and Dexamethasone in Patients with Newly Diagnosed, Untreated Multiple Myeloma Untreated Multiple Myeloma. https://news.bms.com/press-release/corporatefinancial-news/bristol-myers-squibb-reports-primary-results-eloquent-1-study-.

[79] Ribrag V., Avigan D.E., Green D.J., Wise-Draper T., Posada J.G., Vij R., Zhu Y., Farooqui M.Z.H., Marinello P., Siegel D.S. (2019). Phase 1b trial of pembrolizumab monotherapy for relapsed/refractory multiple myeloma: KEYNOTE-013. Br. J. Haematol..

[80] Mateos M.-V., Blacklock H., Schjesvold F., Oriol A., Simpson D., George A., Goldschmidt H., Larocca A., Chanan-Khan A., Sherbenou D. (2019). Pembrolizumab plus pomalidomide and dexamethasone for patients with relapsed or refractory multiple myeloma (KEYNOTE-183): A randomised, open-label, phase 3 trial. Lancet Haematol..

[81] Usmani S.Z., Schjesvold F., Oriol A., Karlin L., Cavo M., Rifkin R.M., Yimer H.A., LeBlanc R., Takezako N., McCroskey R.D. (2019). Pembrolizumab plus lenalidomide and dexamethasone for patients with treatment-naive multiple myeloma (KEYNOTE-185): A randomised, open-label, phase 3 trial. Lancet Haematol..

[82] Bezman N.A., Jhatakia A., Kearney A.Y., Brender T., Maurer M., Henning K., Jenkins M.R., Rogers A.J., Neeson P.J., Korman A.J. (2017). PD-1 blockade enhances elotuzumab efficacy in mouse tumor models. Blood Adv..

[83] Bezman N.A., Kinder M., Jhatakia A.D., Mattson B.K., Pizutti D., Thompson E.W., Capaldi D.A., Mendonca M.W., Anandam A., Dhar G. Antitumor activity associated with dual targeting of CD38 and programmed death-1 (PD-1) pathways in preclinical models. Proceedings of the Cancer Research; American Association for Cancer Research (AACR).

[84] Carpenter R.O., Evbuomwan M.O., Pittaluga S., Rose J.J., Raffeld M., Yang S., Gress R.E., Hakim F.T., Kochenderfer J.N. (2013). B-cell maturation antigen is a promising target for adoptive T-cell therapy of multiple myeloma. Clin. Cancer Res..

[85] Ryan M.C., Hering M., Peckham D., McDonagh C.F., Brown L., Kim K.M., Meyer D.L., Zabinski R.F., Grewal I.S., Carter P.J. (2007). Antibody targeting of B-cell maturation antigen on malignant plasma cells. Mol. Cancer.

[86] Abdallah A.-O.A., Hoffman J.E., Schroeder M.A., Jacquemont C., Li H., Wang Y., Van Epps H., Campbell M.S. (2019). SGNBCMA-001: A phase 1 study of SEA-BCMA, a non-fucosylated monoclonal antibody, in subjects with relapsed or refractory multiple myeloma. J. Clin. Oncol..

[87] Bruins W.S.C., Zweegman S., Mutis T., van de Donk N.W.C.J. (2020). Targeted Therapy With Immunoconjugates for Multiple Myeloma. Front. Immunol..

[88] Tai Y.T., Mayes P.A., Acharya C., Zhong M.Y., Cea M., Cagnetta A., Craigen J., Yates J., Gliddon L., Fieles W. (2014). Novel anti-B-cell maturation antigen antibody-drug conjugate (GSK2857916) selectively induces killing of multiple myeloma. Blood.

[89] De Oca M.R., Bhattacharya S., Vitali N., Patel K., Kaczynski H., Shi H.Z., Blackwell C., Seestaller-Wehr L., Cooper D., Jackson H. (2019). The anti-bcma antibody-drug conjugate gsk2857916 drives immunogenic cell death and immune-mediated anti-tumor responses, and in combination with an ox40 agonist potentiates in vivo activity. HemaSphere.

[90] Zhao H., Atkinson J., Gulesserian S., Zeng Z., Nater J., Ou J., Yang P., Morrison K., Coleman J., Malik F. (2018). Modulation of macropinocytosis-mediated internalization decreases ocular toxicity of antibody–drug conjugates. Cancer Res..

[91] Caraccio C., Krishna S., Phillips D.J., Schürch C.M. (2020). Bispecific Antibodies for Multiple Myeloma: A Review of Targets, Drugs, Clinical Trials, and Future Directions. Front. Immunol..

[92] Cho S.-F., Lin L., Xing L., Wen K., Yu T., Hsieh P.A., Li Y., Munshi N.C., Wahl J., Matthes K. (2019). AMG 701 Potently Induces Anti-Multiple Myeloma (MM) Functions of T Cells and IMiDs Further Enhance Its Efficacy to Prevent MM Relapse In Vivo. Blood.

[93] Kim E.B., Harrington C., Yee A., O’Donnell E., Branagan A., Burke J., Raje N. (2019). Practical considerations and role of Daratumumab retreatment for relapsed refractory Multiple Myeloma. Clin. Lymphoma Myeloma Leuk..

[94] D’Agostino M., Mina R., Gay F. (2020). Anti-CD38 monoclonal antibodies in multiple myeloma: Another cook in the kitchen?. Lancet Haematol..

[95] Fedyk E.R., Zhao L., Koch A., Smithson G., Estevam J., Chen G., Lahu G., Roepcke S., Lin J., Mclean L. (2020). Safety, tolerability, pharmacokinetics and pharmacodynamics of the anti-CD38 cytolytic antibody TAK-079 in healthy subjects. Br. J. Clin. Pharm..

[96] Saltarella I., Desantis V., Melaccio A., Solimando A.G., Lamanuzzi A., Ria R., Storlazzi C.T., Mariggiò M.A., Vacca A., Frassanito M.A. (2020). Mechanisms of Resistance to Anti-CD38 Daratumumab in Multiple Myeloma. Cells.

[97] Ghose J., Viola D., Terrazas C., Caserta E., Troadec E., Khalife J., Gunes E.G., Sanchez J., McDonald T., Marcucci G. (2018). Daratumumab induces CD38 internalization and impairs myeloma cell adhesion. Oncoimmunology.

[98] Nijhof I.S., Groen R.W.J., Lokhorst H.M., Van Kessel B., Bloem A.C., Van Velzen J., De Jong-Korlaar R., Yuan H., Noort W.A., Klein S.K. (2015). Upregulation of CD38 expression on multiple myeloma cells by all-trans retinoic acid improves the efficacy of daratumumab. Leukemia.

[99] García-Guerrero E., Gogishvili T., Danhof S., Schreder M., Pallaud C., Pérez-Simón J.A., Einsele H., Hudecek M. (2017). Panobinostat induces CD38 upregulation and augments the antimyeloma efficacy of daratumumab. Blood.

[100] Madduri D., Usmani S.Z., Jagannath S., Singh I., Zudaire E., Yeh T.-M., Allred A.J., Banerjee A., Goldberg J.D., Schecter J.M. (2019). Results from CARTITUDE-1: A Phase 1b/2 Study of JNJ-4528, a CAR-T Cell Therapy Directed Against B-Cell Maturation Antigen (BCMA), in Patients with Relapsed and/or Refractory Multiple Myeloma (R/R MM). Blood.

[101] D’Agostino M., Zaccaria G.M., Ziccheddu B., Rustad E.H., Genuardi E., Capra A., Oliva S., Auclair D., Yesil J., Colucci P. (2020). Early Relapse Risk in Patients with Newly Diagnosed Multiple Myeloma Characterized by Next-generation Sequencing. Clin. Cancer Res..

